# Standardizing shear wave elastography for sarcopenia assessment: A necessary step forward

**DOI:** 10.1111/ggi.70147

**Published:** 2025-08-14

**Authors:** Yi‐Ching Chu, Chao‐Chun Huang

**Affiliations:** ^1^ Department of Ophthalmology Far Eastern Memorial Hospital New Taipei City Taiwan; ^2^ Department of Physical Medicine and Rehabilitation Far Eastern Memorial Hospital New Taipei City Taiwan; ^3^ Department of Physical Therapy and Assistive Technology National Yang Ming Chiao Tung University Taipei Taiwan

Dear Editor,

We read with great interest the recent systematic review by Gutiu *et al*. in *Geriatrics & Gerontology International*, titled “Muscle elasticity variations in assessing age‐related changes in adults”.^1^ The authors are to be commended for their excellent work in synthesizing the complex and often contradictory literature on the use of shear wave elastography (SWE) to assess age‐related muscle changes.

The most cautionary conclusion of the review is the authors' inability to carry out a meta‐analysis due to “substantial variability in methodologies” across studies.^1^ This is not a limitation of the review, but a powerful statement on the current state of the field. This finding highlights a systemic issue: methodological inconsistency has become a primary barrier to translating SWE from a research tool to a clinical application, creating a critical “translational gap.” Without standardization, large‐scale validation studies are impossible; without validation, SWE cannot be integrated into clinical guidelines, such as those defined by the European Working Group on Sarcopenia in Older People,^2^ for the diagnosis and monitoring of sarcopenia. This is not an isolated problem; other reviews have similarly noted a “striking lack of consensus” and a “pressing need for developing standardized, validated scanning protocols” in musculoskeletal SWE research.^3^

Gutiu *et al*. clearly outlined the multiple facets of this heterogeneity, which confound the interpretation of results and hinder the establishment of SWE as a reliable biomarker. Key issues include:

1. Divergent definitions of muscle state: Measurements vary dramatically depending on whether the muscle is relaxed, passively stretched or actively contracted. For instance, muscle stiffness increases with age under passive stretch, yet decreases with age during active contraction.^1^ These seemingly contradictory results might reflect different underlying pathophysiological mechanisms. The increase in passive stiffness might be related to changes in the extracellular matrix (e.g. fibrosis and fat infiltration),^4^, ^5^, ^6^, ^7^ whereas the decline in active contraction capacity might stem from the loss of contractile units (sarcopenia) and impaired voluntary activation.^1^ Without standardized protocols, we cannot reliably distinguish these biological signals.
2. Inconsistent selection of anatomical regions: Studies have examined a wide range of muscles, from the lower limbs (e.g. quadriceps, gastrocnemius) to the trunk and upper limbs.^1^ Given that muscle aging is not synchronous throughout the body—with functional decline in the lower limbs typically preceding that in the upper limbs^1^, ^8^—this variability in selection makes cross‐study data integration nearly impossible.
3. Confounding technical parameters and reporting units: Different studies use various ultrasound machines, software, probe frequencies and reporting units (e.g. kilopascals [kPa] vs meters/s [m/s]). These non‐biological technical differences introduce significant variability into the measurements, creating substantial methodological “noise” that can obscure the true “signal” arising from pathological changes.^1^

Therefore, we support and echo the conclusion of Gutiu *et al*. that “refining standardized SWE protocols” is an urgent priority.^1^ We further propose the formation of an international working group composed of experts in geriatrics, radiology, rehabilitation medicine and biomedical engineering/physics. This cross‐disciplinary collaboration is crucial, as the solution requires not only clinical consensus, but also the deep understanding of the underlying physics of SWE technology that physicists and engineers can provide, thereby bridging the gap between clinical needs and technical reality. The success of similar consensus‐building initiatives in establishing standardized guidelines for liver elastography^9^ and the broader push for structured reporting in radiology^10^ underscore the feasibility and importance of such an endeavor.

The core mission of this working group should be to develop a consensus‐based clinical guideline that standardizes, at a minimum:

- Patient protocol: Standardized body positioning (e.g. joint angles), a clear definition of the “resting state,” and specific procedures for passive stretching (e.g. specific angles of flexion/extension) and active contraction (e.g. a specific percentage of maximal voluntary contraction).
- Acquisition protocol: Recommendations for probe selection, imaging depth, size and placement of the region of interest, and guidance on how to manage technical differences between vendors.
- Reporting protocol: A consensus on reporting units (e.g. recommending the use of shear wave velocity in m/s to avoid assumptions about tissue density) and a requirement for clear documentation of all technical parameters to ensure study reproducibility.^3^

Only through such rigorous standardization can we facilitate multicenter studies and meta‐analyses, establish reliable normative reference values, and ultimately validate the full potential of SWE as a non‐invasive, accessible biomarker in the diagnosis and management of sarcopenia and age‐related muscle decline.

## Funding information

The authors received no specific funding for this work.

## Disclosure statement

The authors declare no conflict of interest.

## Author contributions

Y‐CC conceived the study, carried out the literature search and drafted the manuscript. C‐CH provided critical revision, supervised the project and is the guarantor. Both authors have read and approved the final manuscript.

## Ethics statement

This article is a commentary on a previously published study and does not involve human participants or animals. Therefore, institutional review board approval was not required.

## Data Availability

Data sharing is not applicable to this article, as no new data were created or analyzed in this study.
